# Ionic Polymer-Coated Laccase with High Activity and Enhanced Stability: Application in the Decolourisation of Water Containing AO7

**DOI:** 10.1038/srep08253

**Published:** 2015-02-05

**Authors:** Xiaolin Zhang, Ming Hua, Lu Lv, Bingcai Pan

**Affiliations:** 1 State Key Laboratory of Pollution Control and Resource Reuse, School of the Environment, Nanjing University, Nanjing 210023, P.R. China

## Abstract

Eliminating dyes in environmental water purification remains a formidable challenge. Laccase is a unique, environmentally friendly and efficient biocatalyst that can degrade pollutants. However, the use of laccase for the degradation of pollutants is considerably limited by its susceptibility to environmental changes and its poor reusability. We fabricated a novel biocatalyst (LacPG) by coating polyethylenimine onto the native laccase (Lac) followed by crosslinking with glutaraldehyde. The stability of the resulting LacPG was highly enhanced against pH variations, thermal treatments and provided better long-term storage with a negligible loss in enzymatic activity. Compared to Lac, LacPG exhibited significantly higher decolourisation efficiency in the degradation of a representative azo dye, acid orange 7 (AO7), which resulted from the electrostatic attraction between the coating and AO7. LacPG was separated from the AO7 solution using an ultrafiltration unit. The increased size and modified surface chemistry of LacPG facilitated ultrafiltration and reduced membrane fouling. LacPG exhibited enhanced stability, high catalytic activity and favourable properties for membrane separation; therefore, LacPG could be continuously reused in an enzymatic membrane reactor with a high efficiency for decolourising water containing AO7. The developed strategy appears to be promising for enhancing the applicability of laccase in practical water treatment.

Synthetic dyes are widely used in industrial dyeing and printing processes. The worldwide annual production of synthetic dyes is greater than 700,000 tons^1^. Azo dyes contain an azo group (-N = N-) as a chromophore that is associated with aromatic structures containing functional groups such as –OH and –SO_3_H, and these dyes constitute ≃70% of the dyes that are used worldwide^2^. It has been estimated that 2–50% of the azo dyes that are used in industrial processes undesirably end up in wastewater^3^. The adverse effects from various azo dyes and their metabolites include water colouration, water toxicity, carcinogenicity and mutagenicity^4^. Azo dyes are xenobiotic compounds that are resistant to biodegradation in conventional aerobic treatment processes^5^. It is known that ≃90% of azo dyes pass through activated sludge sewage treatment plants and persist as residues in the effluents^6^,^7^. Numerous processes have been developed to minimise the adverse effects of azo dyes on water quality, including adsorption and advanced oxidation processes^8^. However, the majority of the available techniques are generally still far from being used in practical applications because of efficiency and/or economic limitations.

Laccase (polyphenoloxidase, EC 1.10.3.2) is a multicopper oxidase that is secreted by various plants and fungal species^9^, and it can oxidise phenolic moieties and amine compounds^10^. The oxidation range of laccase can be expanded to non-phenolic substrates through combination with redox mediators such as ABTS (2,2′-azinobis(3-ethyl benzth-iazoline-6-sulfonate))^11^, HBT (1-hydroxybenzotriazole)^12^ and natural mediators^13^. Laccase is attracting increasing interest for use in wastewater treatment^14^,^15^,^16^, including effluents containing dyes^17^,^18^,^19^,^20^,^21^. However, the direct use of laccase in wastewater treatment is not an attractive option because this enzyme is expensive and has poor reusability, which is directly related to its high solubility and weak stability. Changes in environmental conditions (e.g., pH and temperature) and long storage time have been shown to deactivate this enzyme.

To apply laccase in practical applications, stable and reusable laccase derivatives must be prepared. The immobilisation of laccase onto/into solid supports is one of the most common available preparation strategies^22^,^23^,^24^,^25^. As expected, this immobilisation improves the stability of laccase^23^,^26^. Laccase can also be readily recycled using a simple solid-liquid separation^26^,^27^ or magnetic separation^28^. Unfortunately, a severe loss of enzymatic activity inevitably occurs because of the high diffusional limitation of the substrates^23^,^26^,^31^ and because of the considerable quantity of unavailable laccase following immobilisation.

The stabilisation of enzymes in soluble form has recently been developed as a promising alternative to the stabilisation of enzymes by solid hosts. Without the spatial limitations of a solid host, the stabilised enzyme can be sufficiently effective for biocatalysis, and the mass transfer of the substrates is significantly improved^29^,^30^,^31^,^32^. Physically coating enzymes with ionic polymers is one of the most attractive strategies for obtaining stabilised enzymes in soluble form^29^,^30^,^31^,^32^,^33^,^34^,^35^,^36^,^37^. This approach features simple operation and has little effect on the protein conformation, and hence, minimal effects on the enzymatic activity. However, without further treatment, directly coated enzymes are unstable in aqueous systems^31^.

In the present study, we developed a simple strategy for stabilising laccase using polyethylenimine (PEI) coatings followed by crosslinking using glutaraldehyde (GA). PEI has one of the highest amino-group densities of aminated polymers, which enables PEI to be coated onto the surface of laccase via ion exchange^36^. Further crosslinking is used to make the PEI coating process irreversible in solution. Considering the widespread use of membrane techniques in wastewater treatment, we designed a simple enzymatic membrane reactor and incorporated ultrafiltration as the key unit for separating and recycling the resulting laccase composite for use in cyclic water decolourisation of a representative azo dye, acid orange 7 (AO7).

## Results

All of the following results were obtained from triplicate experiments, and the error bars show the standard deviations, unless stated otherwise.

### Fabrication of PEI-coated Laccase (LacPG)

**Figure 1:**
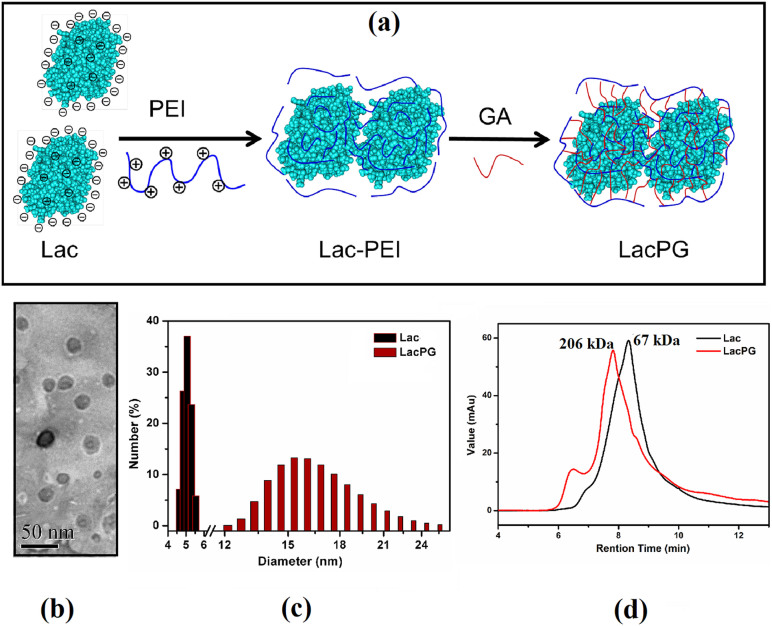
(a) Schematic of LacPG fabrication, (b) TEM image of LacPG, and (c) size and (d) molecular weight distribution of Lac and LacPG determined by DLS and SEC, respectively; error bars show standard deviations for at least triplicate measurements.

**Figure 2:**
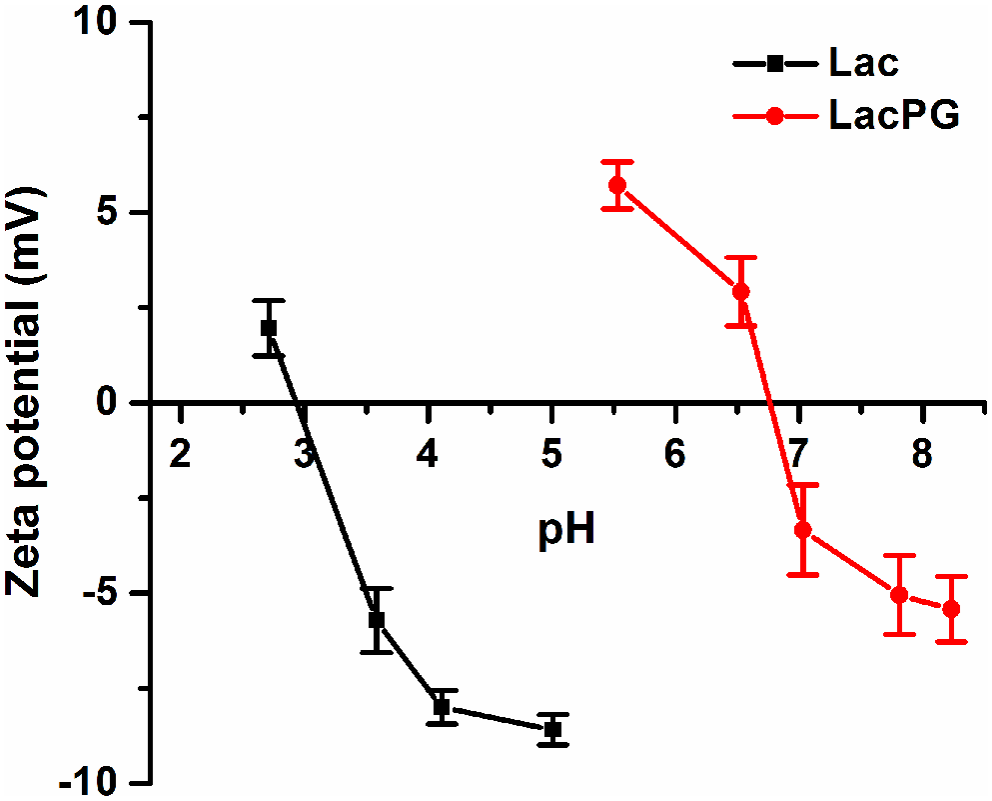
Zeta potentials of Lac and LacPG; error bars show standard deviations for at least five measurements.

Figure 1(a) presents a schematic that illustrates the fabrication of LacPG. Briefly, native laccase (denoted as Lac) was modified by PEI to produce Lac-PEI, which was then crosslinked by GA to produce the novel biocatalyst LacPG. UV-Vis spectra (Supplementary Fig. S1) revealed that a significant increase in the absorption intensity occurred for LacPG from 310 to 550 nm compared to a mixture of Lac and PEI-G (which corresponded to the solution of PEI that was crosslinked by GA) at the same concentration level, indicating that LacPG was a new product rather than a simple mixture. A topographic image of LacPG is shown in Fig. 1(b), in which LacPG is observed as shadows with an average size of approximately 20 nm. The size distributions of Lac and LacPG were measured by dynamic light scattering (DLS) (Fig. 1(c)), which indicated a Lac diameter within 4–6 nm, in agreement with the previously reported value for *Trametes versicolor* laccase^38^; however, a larger average diameter of 15–17 nm was observed for LacPG, which was consistent with the results shown in Fig. 1(b). Figure 1(d) shows the molecular weight (Mw) of Lac and LacPG determined by size exclusion chromatography (SEC). Lac molecules had a molecular weight of 67 kDa, consistent with the previously reported Mw of laccase from *Trametes versicolor* of 69 kDa^38^, whereas most of the LacPG molecules had larger Mw of 206 kDa. In addition to the aforementioned changes in size or molecular weight, the zeta potentials of Lac and LacPG (see Fig. 2) revealed that the isoelectric point (PI) for LacPG (≃6.8) was considerably higher than that for Lac (≃2.9), primarily because the PEI coating on the Lac molecule contained an abundance of positively charged groups. The surface chemistry alteration of LacPG grant more opportunity for anionic substrate to react with LacPG through enhanced electrostatic attraction, which is favourable for decolourisation of water containing anionic azo dye, as elucidated below.

### Activity of LacPG

After fabrication, the apparent activity of 7.69 ± 0.31 U·mg^−1^ for Lac changed to 7.25 ± 0.69 U·mg^−1^ for LacPG (per mg laccase protein, data not shown). The kinetic constants (K_m_, the Michaelis–Menten constants) and the turnover numbers (k_cat_) for Lac and LacPG on two of the most widely used substrates, ABTS and 2,6-dimethoxyphenol (DMP), were also determined using the Michaelis-Menten equation and are listed in Table 1. Compared to Lac, LacPG had a slightly smaller k_cat_ and a higher K_m_ for both ABTS and DMP, which decreased its catalytic efficiency (k_cat_/K_m_). The effects of pH and temperature on the activities of Lac and LacPG were examined, and the results are shown in Supplementary Fig. S2. LacPG and Lac exhibited similar responses in activity to variations in pH and temperature. An increase in pH from 2.5 to 4.0 or in temperature from 25°C to 55°C enhanced the activities of both Lac and LacPG, whereas further increases in pH or temperature decreased the corresponding activities. It is well known chloride ion is a common inhibitor for fungal laccase^39^; the effect of chloride concentration on the activities of Lac and LacPG were determined at NaCl concentration range from 20 to 500 mM under otherwise identical conditions and the results were shown in Supplementary Fig. S3. Both Lac and LacPG exhibited decreased activities in the presence of chloride ions. LacPG was observed with higher activity retention than Lac, e.g., 40 mM chloride ions caused ≃40% and ≃25% activity losses for Lac and LacPG, respectively.

### Stability of LacPG

**Figure 3:**
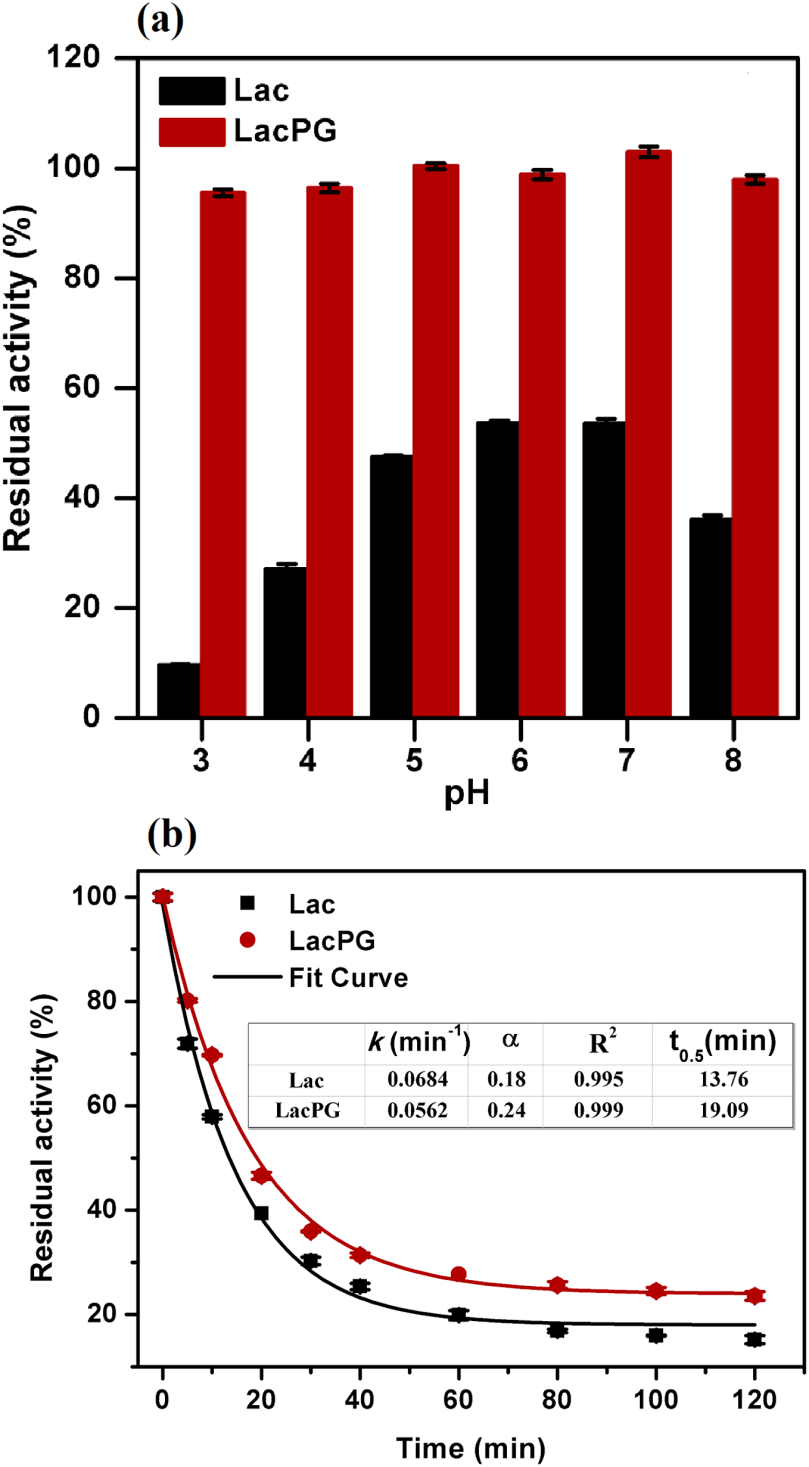
(a) Effect of pH variation (25°C, incubation for 96 h) and (b) thermal treatment (pH 7.0, 60°C) on the residual activity of Lac and LacPG; error bars show standard deviations for triplicate measurements.

In general, enzymatic biocatalysts are susceptible to variations in pH and temperature during the catalytic degradation of organic pollutants, which greatly limits their practical application to water treatment. Thus, we examined the performance of LacPG under variations in pH and temperature. Figure 3(a) shows that a negligible loss in the activity of LacPG was observed after incubating the system over a pH range between 3 and 8 for 96 h at 25°C, whereas Lac lost ca. 50–90% of its initial activity. A slower deactivation rate was observed in the thermal study for LacPG than for Lac when the systems were incubated at 60°C (Fig. 3(b)). The thermal stability was quantitatively evaluated by determining the thermal parameters using the following simplified deactivation model^40^.
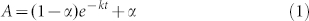

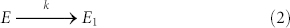


This model involves two enzymatic states (E and E_1_, see Equation (2)), where *k* is a first-order deactivation rate coefficient and *α* is the ratio of the specific activities of these states (activity of E_1_/activity of E). In Equation (1), *A* denotes the residual activity and *t* is the time (in units of min). The half-life of laccase at 60°C (*t_0.5_*), i.e., the period of time required for a substance to decay to half of its initial concentration, was calculated from Equation (1) using the estimated parameters with *A* set to 0.5. The *t_0.5_* of LacPG was longer than that of Lac (19.09 min versus 13.76 min, respectively), suggesting that the thermal stability of LacPG was enhanced.

**Figure 4:**
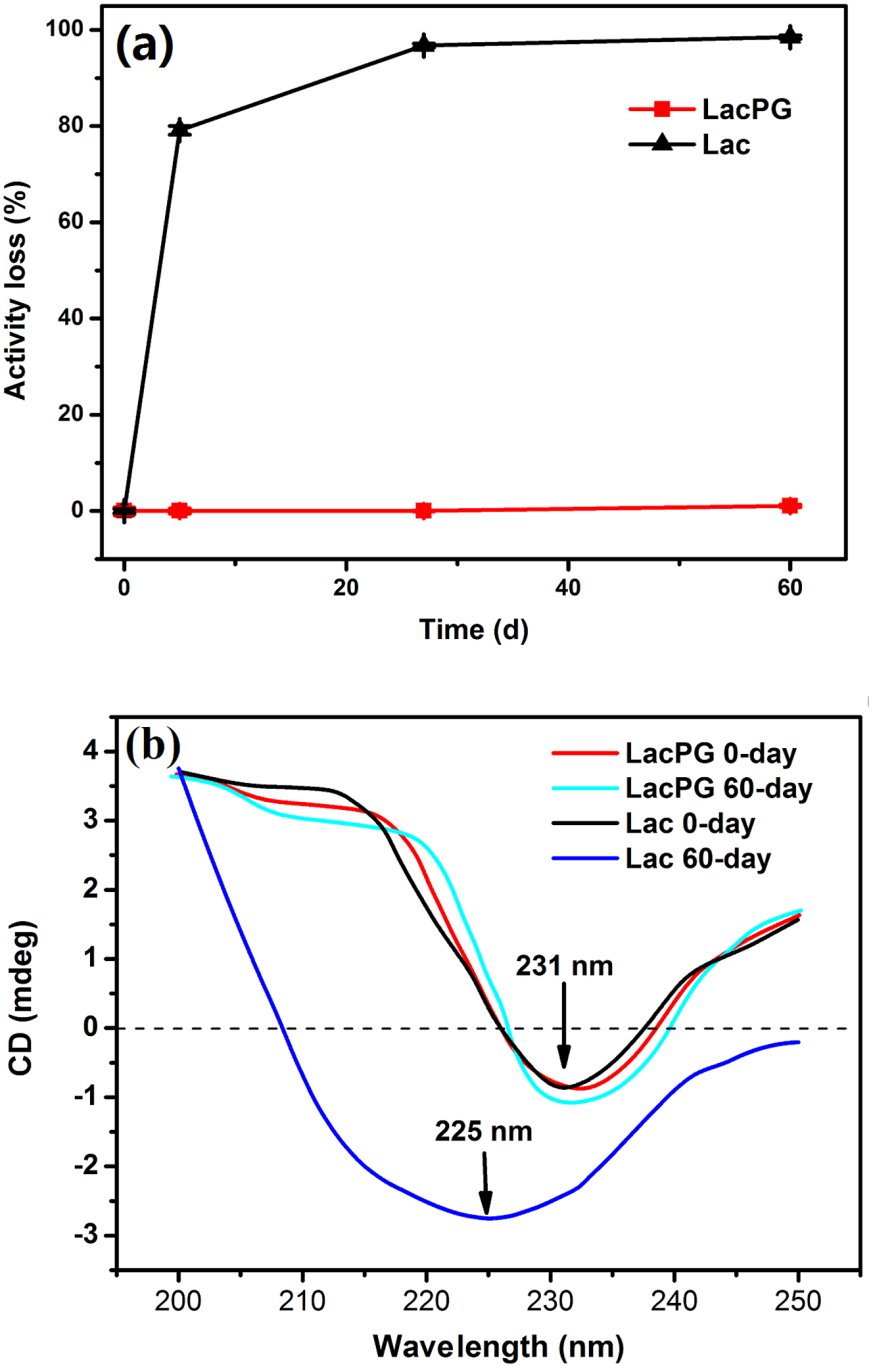
(a) Storage stabilities of Lac and LacPG and (b) far-UV CD spectra of Lac and LacPG before and after 60 days of storage (pH 7.0, 40°C); error bars show standard deviations for at least three measurements; all of the presented CD spectra were averaged over at least five scans, and the contribution from the background buffer solution was subtracted.

Good storage stability is an essential requirement for a biocatalyst to be used in industrial applications. It is well known that high temperatures are detrimental to the storage of enzymes. Thus, the storage stabilities of Lac and LacPG were examined at 40°C, which could be considered to be scorching weather, and the results are shown in Fig. 4(a). Lac lost ca. 80% and 100% of its activity after 5 days and 60 days of storage, respectively, whereas no loss in activity was detected for LacPG. We explored the secondary conformations of Lac and LacPG by recording their circular dichroism (CD) spectra before and after 60 days of storage (Fig. 4(b)). The crosslinked PEI coatings had little effect on the secondary conformation of Lac, and the CD spectrum of fresh Lac had a similar shape to that of fresh LacPG, in which the negative peak at approximately 231 nm was assigned to the arrangements of β-sheets in the secondary structure^41^. After 60 days of storage, there was a considerable change in the CD spectrum of Lac, e.g., the negative peak at ≃231 nm shifted to ≃225 nm and increased in intensity, indicating a conformational change in Lac resulting from storage. However, 60 days of storage had a negligible effect on the CD spectrum of LacPG, and only a slight increase in the intensity of the negative peak at ≃231 nm was observed, indicating that the secondary structure of LacPG was stable.

### Decolourisation of water containing acid orange 7 (AO7)

**Figure 5:**
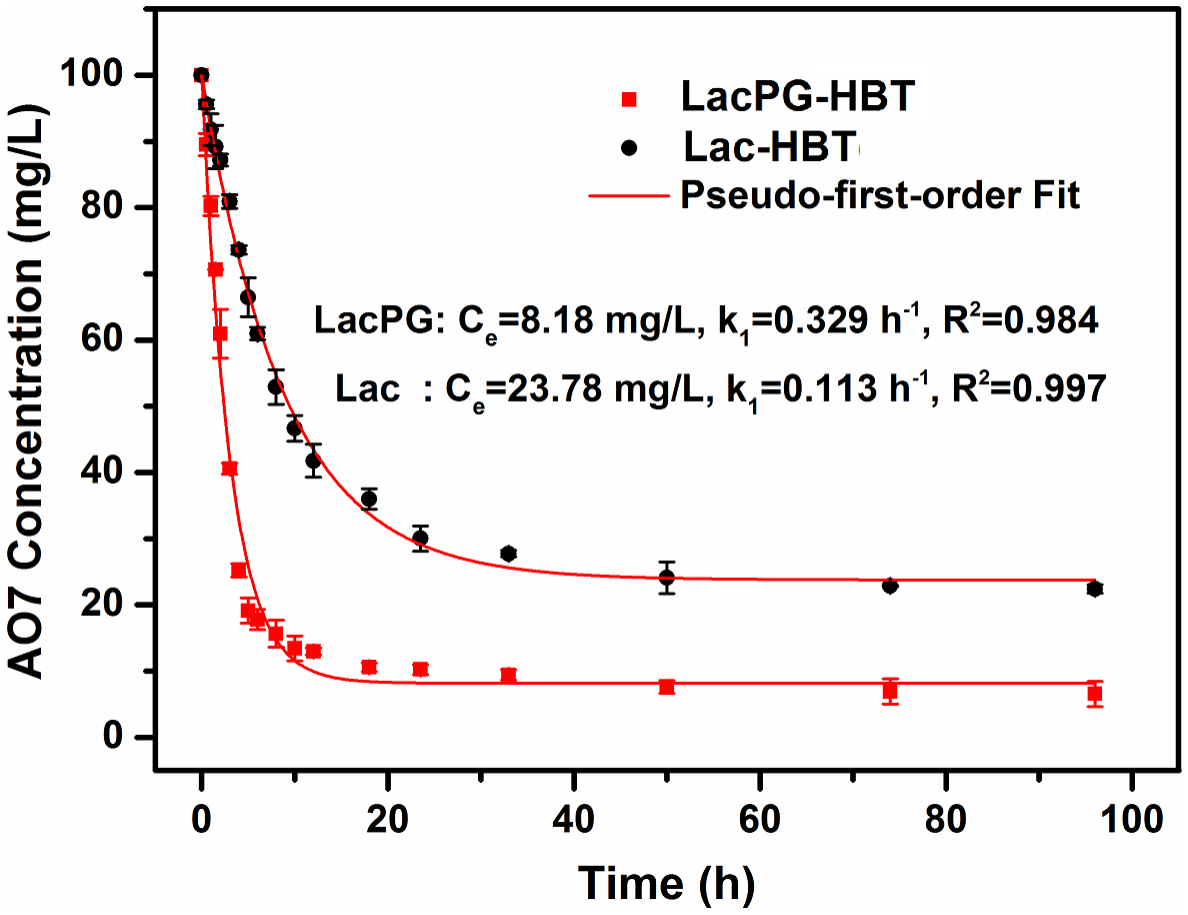
Decolourisation of AO7 using Lac and LacPG (pH 5.0, 25°C) in the presence of HBT; error bars show standard deviations from triplicate measurements.

A representative azo dye, AO7 (its structure was schemed in Supplementary Fig. S4), was used to test the decolourisation ability of LacPG in the presence of 0.1 mM HBT. The kinetic data were fitted using the following pseudo-first-order model^42^:
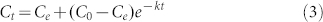
where *C_t_* is the dye concentration (in units of mg·L^−1^), *t* is the time (in units of h), *k* is the first-order reaction rate constant (in units of h^−1^), *C_o_* is the initial dye concentration (100 mg·L^−1^, in this case), and *C_e_* is the equilibrium concentration of the tested dye (mg·L^−1^). Figure 5 shows that the decolourisation rate of LacPG was ca. 3-fold higher than that of Lac (0.33 h^−1^ versus 0.11 h^−1^) and that the equilibrium AO7 concentration for LacPG was only ca. 0.25-fold that for Lac (8.2 mg·L^−1^ versus 23.8 mg·L^−1^). These results suggested that the PEI coating on LacPG accelerated the degradation of AO7 and improved the decolourisation efficiency of LacPG.

### Decolourisation of water containing malachite green (MG)

To further explore the role of the PEI coating, a cationic dye, MG (its structure was schemed in Supplementary Fig. S4), was degraded for comparison with the degradation of AO7 under otherwise identical conditions. The best fit to the kinetic data was obtained using the following pseudo-second-order model^42^:
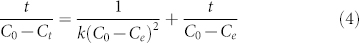
where *C_t_* is the dye concentration (in units of mg·L^−1^), *t* is the time (in units of h), *k_1_* or *k* is the second-order reaction rate constant (in units of L·mg^−1^·h^−1^), *C_o_* is the initial dye concentration (100 mg·L^−1^, in this case), and *C_e_* is the equilibrium concentration of the tested dye (mg·L^−1^). The mechanism of laccase-meditated decolourisation strongly depends on the structure of the dye^50^. It has been reported that the biodegradation of azo dyes follows first-order kinetics^51^, whereas that of triphenylmethane dyes follows second-order kinetics^52^. In contrast to the results obtained for AO7, slower decolourisation kinetics (Supplementary Fig. S5) and a lower decolourisation efficiency (Supplementary Fig. S5) were observed using LacPG than using Lac.

### Enzymatic membrane reactor for cyclic decolourisation

**Figure 6:**
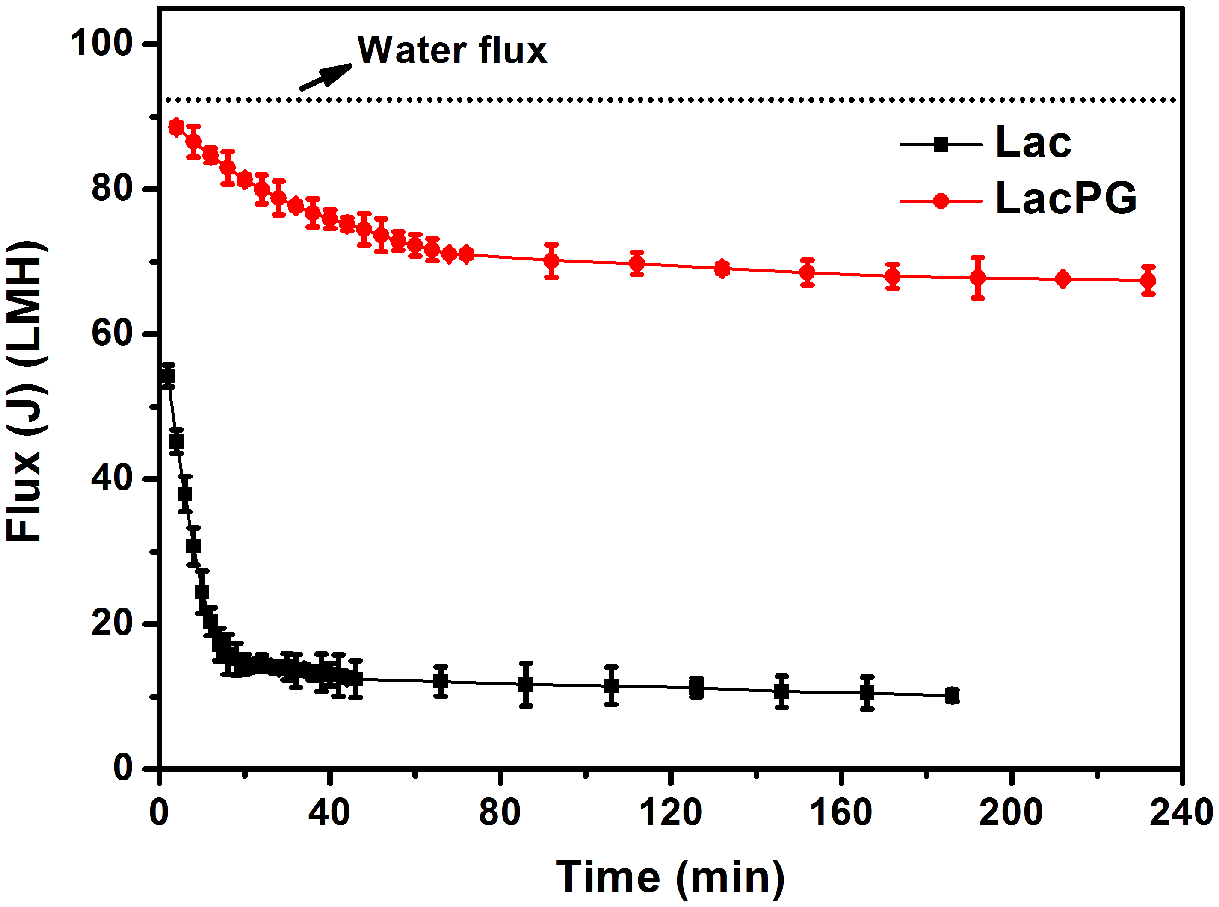
Flux change of ultrafiltration membrane (MWCO = 30 kDa) for deionised water, Lac and LacPG (25°C, pressure = 0.1 MPa); error bars shows standard deviations from triplicate measurements.

An enzymatic membrane reactor was designed to recycle the enzyme for the decolourisation of the AO7 solution (see Supplementary Fig. S6). Supplementary Figure S7 shows that more than 99% of the LacPG or Lac was rejected by the ultrafiltration polyethersulphone (PES) membrane with a nominal molecular weight cut-off (MWCO) of 30 kDa. Using membranes with MWCOs of 50 kDa and 100 kDa resulted in a significant decrease in the Lac rejection rate to ca. 92% and ca. 12%, respectively, whereas that for LacPG remained at ca. 99% and ca. 95%, respectively. It is reasonable because of the increased size of LacPG relative to Lac as shown in Fig. 1. Prior to performing the decolourisation assay, the performances of Lac and LacPG on membrane fouling were examined because membrane fouling is one of the major obstacles in maintaining efficient performance of an ultrafiltration membrane^43^. Figure 6 shows the permeate flux that was recorded for the membrane with a MWCO of 30 kDa under a constant pressure of 0.1 MPa. The deionised water flux was constant at ca. 92 L·m^−2^·h^−1^ (LMH). The deionised water flux initially decreased significantly over 20 min for Lac and over 80 min for LacPG. Compared with the flux of deionised water, the initial LacPG flux decreased slightly to ca. 89 LMH and that for Lac decreased to ca. 54 LMH. The LacPG flux at the end of the experiment (ca. 67 LMH) suggested that the membrane was moderately fouled. In contrast, severe membrane fouling was observed using Lac (the Lac flux decreased to ca. 10 LMH).

**Figure 7:**
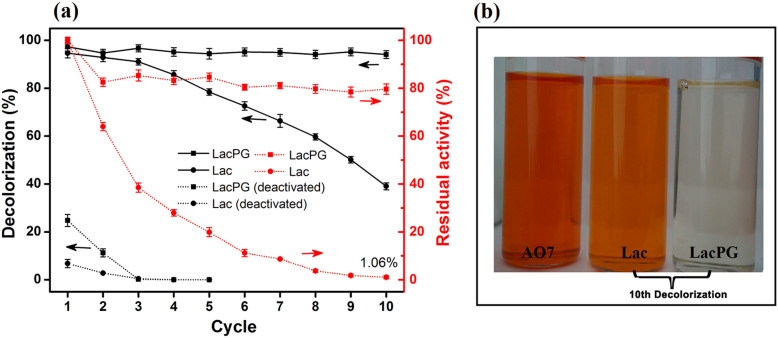
Cyclic Lac or LacPG-mediated decolourisation of AO7 solution: (a) decolourisation efficiency for active or deactivated biocatalysts (deactivated at 60°C for at least 24 h) and residual enzymatic activity and (b) fresh AO7 solution (100 mg·L^−1^) and effluents from the 10th cyclic run. Biodegradation of 100 mg·L^−1^ AO7 phosphate buffer solution (pH 5.0, 0.05 M) containing 0.1 mM HBT and LacPG or native laccase (0.1 mg laccase protein per mL, ca. 0.8 U·mL^−1^) at 25°C; LacPG or Lac were recycled for continuous runs; ultrafiltration: pressure = 0.1 MPa, 25°C; error bars show standard deviations for triplicate measurements.

The cyclic decolourisation results for Lac and LacPG are shown in Fig. 7. Heat deactivation (60°C for at least 24 h) was used to estimate the effects of AO7 absorption by Lac or LacPG in the enzymatic membrane reactor under otherwise identical conditions. The deactivated LacPG and Lac removed ca. 25% and 7% of AO7 by adsorption in the first run, respectively, whereas the decolourisation efficiency sharply decreased to ca. 0% within three cyclic runs. When using either Lac or LacPG, the decolourisation efficiency was as high as ca. 94% during the first cyclic run. After ten cyclic runs, the decolourisation efficiency of Lac dramatically decreased to ca. 39%, and the corresponding laccase activity decreased to ≃1% (≃8.5 U·L^−1^). In contrast, after ten cyclic runs, the activity of LacPG remained at >80% of its initial activity. Thus, LacPG consistently maintained its decolourisation efficiency (ca. 94%) in the enzymatic membrane reactor even after ten cyclic runs.

## Discussion

Both the molecular diameter (Fig. 1(b), Fig. 1(c)) and the molecular weight (Fig. 1(d)) of LacPG after fabrication indicated that the size of LacPG was significantly increased relative to that of Lac. A similar change in size has been observed for other enzymes that have been stabilised in soluble form by prior chemical modification^34^,^35^. The change in the size of LacPG resulted from the thickness of the PEI coating followed by crosslinking among Lac and PEI molecules.

The catalytic efficiency (k_cat_/K_m_) of LacPG was inhibited relative to that of Lac, primarily because the K_m_ of LacPG was higher than that of Lac (Table 1), indicating that the affinity between the catalytic centre and the substrates was reduced by modifying Lac. This result occurred because the PEI coating restricted laccase from accessing the substrates (ABTS or DMP) for further reaction. However, the catalytic efficiency of LacPG was still higher than that of enzymes that have been immobilised inside the porous solid hosts, which have presented considerably higher K_m_ values^23^,^40^,^44^,^45^. Note that LacPG had a slightly lower turnover number (k_cat_) than Lac, indicating that the available active sites for laccase were negligibly affected by the PEI coating, whereas the immobilised Lac typically had substantially fewer sites than the native Lac^23^,^45^. The change in k_cat_ was consistent with the slight decrease in the specific activity of LacPG. The slight decrease in both k_cat_ and the specific activity of LacPG could be attributed to the small effect of the PEI coatings on the conformation of Lac, as shown in Fig. 4(b), potentially because the flexible PEI coating that was formed by gentle adsorption and subsequent crosslinking adapted to the laccase molecule rather than forcing the laccase molecule to alter its conformations. In realistic, dye effluents often contain remarkable concentration of sodium chloride, and it is an important work to develop laccase bearing activity at high concentration of chloride ions. The activities determined at various concentrations of sodium chloride suggested LacPG less sensitive to chloride ions than Lac (Supplementary Fig. S3); it is deduced that the ion-exchange effect of PEI coating has inhibited the accessibility of chloride ions to the activity site of LacPG.

In addition to its high activity, LacPG was observed to exhibit enhanced stability against pH variations, thermal treatments and long-term storage (Fig. 3 and Fig. 4). The dramatic change that was observed upon crosslinking was that the decrease in the activity of LacPG was negligible for incubation under pH values ranging between 3 and 8 or for storage at 40°C for 60 days. This considerable improvement in stability primarily resulted from the significant conformational rigidity offered by the irreversible PEI coating, as shown in Fig. 4(b). Moreover, the PEI coating was protonated in an acidic environment, in which the protonated amino groups would have decreased the hydrogen ion concentration near the LacPG surface because of co-ion exclusion^46^,^47^,^48^; this effect may have also contributed to stabilising LacPG against pH variations. We reiterate that the excellent stability of LacPG against pH variations and long-term storage was achieved without compromising the catalytic activity of the enzyme.

Laccase can oxidise several particular small molecules known as mediators, these oxidized mediators can in turn attack target molecules that would not normally be a substrate for the enzyme^14^. These mediators have increased the potential application scope of laccase tremendously^49^. In this study, HBT was added as mediator. In decolourising water containing AO7, LacPG exhibited considerably faster kinetics and a higher decolourisation efficiency than Lac (Fig. 5); in decolourising water containing MG, slower decolourisation kinetics (Supplementary Fig. S5) and a lower decolourisation efficiency (Supplementary Fig. S5) were observed using LacPG than using Lac. We hypothesise that the electrostatic attraction between the PEI coating and the AO7 may have played a key role in improving the performance of LacPG.

**Figure 8:**
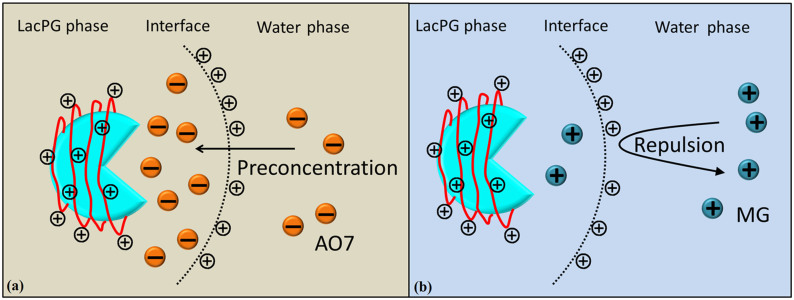
Potential role of PEI coatings on LacPG decolourisation of (a) AO7 and (b) MG.

Figure 8 shows that the PEI coating created a new surface chemistry for LacPG: the LacPG surface was positively charged at pH 5, whereas the Lac surface was negatively charged at this pH. Thus, the electrostatic attraction would cause anionic AO7 molecules to concentrate near the surface of the positively charged LacPG. Similarly, a lower concentration of cationic MG was expected near the LacPG surface than in the bulk solution. The dye concentration distribution near the LacPG surface affected the ability of LacPG to degrade the targeted dyes.

High concentration of sodium chloride plays an adverse role in water purification techniques. The effects of high concentration of sodium chloride on AO7 decolourisation were examined at 0.5 M sodium chloride under otherwise identical conditions. It is obvious 0.5 M sodium chloride inhibited the decolourisation of water containing AO7 severely for both Lac and LacPG from Supplementary Fig. S8. The decolourisation ability of Lac was inhibited completely, while LacPG still transformed ≃15% dye. Observed from Supplementary Fig. S3, LacPG had similar activity retention with Lac at 0.5 M NaCl conditions. The difference in decolourisation between LacPG and Lac was largely arising from the electrostatic attraction between PEI coating and AO7 molecules as mentioned before. Although far away from the standard of practical applications, LacPG implied a potential approach to improve the chloride tolerance of fungal laccase.

In absence of HBT, decolourisation of water containing AO7 was determined under otherwise identical conditions. The results in Supplementary Fig. S9(a) confirmed the decolourisation efficiency declined significant to ≃19% and ≃34% for Lac and LacPG, respectively. It is reasonable because the redox potential of laccase from *Trametes versicolor* is relative low (0.45–0.8 eV)^9^. Because of the electrostatic attraction between PEI coating and AO7, LacPG exhibited significant higher decolourisation efficiency relative to Lac, The high performance liquid chromatography (HPLC) results in Supplementary Fig. S9(b) suggested AO7 undergo different degradation pathways when decolourised by LacPG in presence and in absence of HBT, and the presence of HBT made the degradation much more complicated. The underlying mechanism of the differences caused by HBT and its potential impacts on the applications of LacPG require further study.

The ultrafiltration membrane was clearly more rapidly and severely fouled during the Lac separation than during the LacPG separation (Fig. 6). Xiao et al. investigated the fouling of an ultrafiltration membrane and found a strong correlation between the fouling resistance and the small molecule content and the zeta potential of the solution, i.e., the higher the concentration of small molecules or the more negative was the charge density of the solution, the more severe was the fouling^43^. Small molecules are easier to pass into membrane pore and cause pore blockage. Also, the number of small molecules is larger than big ones under the same conditions, which means that they could reduce the effective filtration area significantly. The electrostatic interaction between negative solution and PES membrane (it is negative at pH 3–8)^53^, will reduce permeability through the membrane due to a strong repulsive interaction. The authors also observed that small molecules had a more significant effect on membrane fouling than the zeta potential of the solution^40^. The LacPG solution had a higher proportion of large molecules than did the Lac solution (Fig. 1(b), Fig. 1(c) and Fig. 1(d)) and a highly positive charge density (Fig. 2); thus, it was reasonable that the LacPG solution performed better than Lac against membrane fouling. The cyclic decolourisation by the enzymatic membrane reactor in Fig. 7 shows that effect of adsorption on the decolourisation process was limited, except in the first 2 cyclic runs. The higher adsorption by LacPG than by Lac primarily resulted from the positively charged coating on the LacPG surface, which also accelerated the degradation of AO7, as previously described. The constant decolourisation efficiency of LacPG over 10 cyclic runs was primarily attributed to the excellent stability, high activity and favourable properties of LacPG for membrane separation, as previously discussed. The cyclic decolourisation assay demonstrated that enzymatic membrane reactors based on LacPG could be an attractive practical strategy for water treatment.

## Methods

### Chemicals and reagents

Laccase from *Trametes versicolor* and branched polyethylenimine (PEI, average Mw ≃ 25000, average Mn ≃ 10000) were purchased from Sigma-Aldrich. Ultrafiltration polyethersulphone (PES) membranes with nominal molecular weight cut-offs (MWCOs) of 30, 50 and 100 kDa were obtained from Millipore (Billerica, MA, USA). All of the other chemicals used were of the highest purity grade commercially available.

### Fabrication of LacPG

The basic processes for the fabrication of LacPG are illustrated in Fig. 1(a). Briefly, Lac was dissolved in a phosphate buffer (pH 7.0, 0.05 M, 25 mL) to obtain a concentration of 2.0 g·L^−1^. Then, the Lac solution was mixed with 25 mL of the phosphate buffer containing PEI (0.1%, w/v) and vibrated at 25°C for 6 h. Afterwards, the unreacted PEI was removed by dialysing the mixed solution against the phosphate buffer using a membrane with a MWCO of 50 kDa at 25°C, which afforded the Lac-PEI solution. The crosslinking reaction was performed by mixing 50 mL of Lac-PEI with 0.10 mL of GA solution (25%, w/v) and vibrating the resulting solution at 25°C for 6 h to produce the LacPG solution. The protein concentration was determined according to the Bradford method using bovine serum albumin (BSA) as the standard protein^54^.

### Determination of laccase activity

The laccase activity was measured using DMP (1 mM) as the enzymatic substrate at 25°C and pH 5.0 (in a 0.05 M phosphate buffer). The molar extinction coefficient of the oxidation product of DMP at 470 nm is 49.6 mM·cm^−1^. The activity was expressed in U, where 1 U is defined as the amount of laccase required to oxidise 1 μmol of DMP in 1 min. A blank test confirmed that the phosphate buffer solution did not have any effect on the laccase activity.

The effects of pH on the activity were determined in a phosphate buffer (pH 2.5–8.0, 0.05 M) at 25°C, and the effects of temperature were assayed in a phosphate buffer (pH 5.0, 0.05 M) at 25–65°C.

The Michaelis-Menten kinetic parameters, K_m_ and V_max_, for the Lac and LacPG were determined by measuring the laccase activity using ABTS or DMP as substrates over a 0.01–0.5 mM range of initial concentrations in a phosphate buffer (pH 5.0, 0.05 M) containing 6.67 × 10^−4^ mg·mL^−1^ (ca. 0.005 U·mL^−1^) protein at 25°C. The changes in absorbance at 420 nm (ε = 36 × 10^3^ M^−1^·cm^−1^) for the ABTS and at 470 nm (*ε* = 49.7 × 10^3^ M^−1^·cm^−1^) for the DMP were recorded for 30 s. The parameter values were obtained by a nonlinear curve fit to a plot of the reaction rate versus the substrate concentration using the software Origin v8.0 (OriginLab Inc., Northampton, USA).

### Stability of LacPG

The storage stabilities of Lac, LacPG, and Lac-PEI were examined. Each biocatalyst (0.10 mL, 1 mg protein per mL) was added to a phosphate buffer (10 mL, pH 7.0, 0.05 M) and maintained at 40°C. Samples were periodically collected for the activity assay over 60 days. Stability against pH was determined by incubating LacPG and Lac (0.1 mL, 1 mg protein per mL) with a phosphate buffer (10 mL, 0.05 M, pH 3.0–8.0) at 25°C for 96 h, and the residual activity was determined at 25°C and pH 5.0. The thermal stabilities of the enzymes were evaluated by incubating LacPG and Lac (0.1 mL, 1 mg protein per mL) with a phosphate buffer (10 mL, 0.05 M, pH 7.0) at 60°C for 2 h, and the residual activity of the enzymes was determined at different intervals (25°C, pH 5.0).

### Decolourisation assay

The decolourisation reactions occurred in phosphate buffers (50 mL, pH 5.0, 0.05 M) containing 0.05 mg·mL^−1^ laccase protein (Lac or LacPG, ca. 0.4 U·mL^−1^), 100 mg·L^−1^ dye (AO7 or MG) and 0.1 mM HBT at 25°C. In the decolourisation assays, the solution was sampled at different time intervals. The dye concentrations were determined by measuring the absorbance of the solutions at 484 nm for AO7 and at 618 nm for MG with an UV-Vis spectrophotometer.

### Ultrafiltration assay

An enzymatic membrane reactor, i.e., a stirred tank reactor coupled to an ultrafiltration membrane (Supplementary Fig. S6), was run for a batch decolourisation process on the AO7 solution. Membrane filtration was conducted in stirred filtration cell (Model 8200, Amicon Corp., Beverly, MA) using a flat sheet membrane (diameter 63.5 mm) at 25°C. Constant-pressure (0.1 MPa) filtration was maintained by gas pressure regulated from a nitrogen cylinder. Prior to the decolourisation assay, the membrane fouling performance was evaluated by measuring the flux of Lac and LacPG for ultrafiltration using a membrane with a MWCO of 30 kDa. Membrane flux was determined by weighting the permeation at timed intervals. The decolourisation was conducted in phosphate buffers (50 mL, pH 5.0, 0.05 M) containing 0.1 mg·mL^−1^ laccase protein (Lac or LacPG, some were heat deactivated at 60°C for at least 24 h when needed, ca. 0.8 U·mL^−1^), 100 mg·L^−1^ dye and 0.1 mM HBT at 25°C. After the batch decolourisation was completed (ca. 12 h), the solution was subjected to ultrafiltration using a membrane with a MWCO of 30 kDa. The next run in the decolourisation assay was initiated by adding the rejected streams to the fresh dye solution containing 0.1 mM HBT. Before starting a new run, the residual laccase activity of the rejected stream was determined.

### Characterisation

UV-vis spectra over the 200–800 nm wavelength range were recorded for phosphate buffer solutions (pH 7.0, 0.05 M) containing 1 mg·mL^−1^ laccase protein (Lac or LacPG) and the solution mixture of PEI and GA (denoted as PEI-G, 0.05% PEI w/v, 0.05% GA v/v). A LacPG sample was dropped onto a carbon-coated copper grid, desiccated in air, and then observed using a high-resolution transmission electron microscope. The size distributions of the Lac and LacPG were measured by dynamic light scattering (DLS) using a particle size analyser. All of the measurements were performed in triplicate, at least.

The molecular weight (Mw) of Lac and LacPG were determined by size exclusion chromatography (SEC, Agilent 1200 series) equipped with PL aquagel-OH Guard column (50 mm × 7.5 mm, 8 μm) and MIXED-M (300 mm × 7.5 mm, 8 μm), and the solution of phosphate buffer saline (PBS) was used as the mobile phase at a flow of 1 mL·min^−1^. A gel filtration markers kit for protein Mw range from 29 kDa to 700 kDa purchased from Sigma-Aldrich were used, including carbonic anhydrase (bovine erythrocytes, 29 kDa), albumin (bovine serum, 66 kDa), alcohol dehydrogenase (yeast, 150 kDa), β-amylase (sweet potato, 200 kDa), apoferritin (horse spleen, 443 kDa) and thyroglobulin (bovine, 669 kDa).

Before measuring the zeta potentials, test solutions containing 0.1 mg·mL^−1^ laccase protein were prepared. The pH of these solutions was adjusted from 2.0 to 10.0 using 0.1 M HCl or NaOH; the solutions were then further stabilised for 12 h via stirring, and the zeta potentials of the solutions were measured using a Zeta-plus 4 instrument (Brookhaven Instrument Corporation, USA). The zeta potential for each sample was determined at least five times.

The circular dichroism (CD) spectra of the Lac and LacPG were recorded using a JASCO J-815 CD spectropolarimeter (Jasco, Japan). The protein concentration in each sample was adjusted to ca. 0.1 mg·mL^−1^. The data points were recorded from 250 to 200 nm at room temperature using a quartz cell with a 0.1-mm path length. All of the spectra that are presented in this study were averaged over at least five scans to improve the signal-to-noise ratio, and the contribution from the background buffer solution was subtracted.

The metabolite of AO7 degraded by LacPG in presence and in absence of HBT were monitored using Dionex U3000 HPLC (USA), equipped with a C18 column (Agilent TC-C18(2), 5 μm, 4.6 × 250 mm). The oven was set as 40°C. Separations were achieved using 0.1% (v/v) acetic acid/methyl alcohol gradient from 95:5 to 5:95 (40 min) after 5 min of isocratic run, with a constant flow rate of 1 mL/min. Eluted substances were detected at 254 nm.

## Author Contributions

B.C.P. and X.L.Z. conceived the original idea, and X.L.Z. and M.H. performed most of the research work. L.L. participated in the characterisation of the biocomposite. All authors participated in the data analysis and discussed the results. B.C.P. and X.L.Z. wrote the paper, and all authors provided their feedback. All authors have read and have approved the manuscript.

## Supplementary Material

Supplementary Information Supplementary Information

## Figures and Tables

**Table 1:** Michaelis-Menten kinetic parameters of Lac and LacPG using ABTS and DMP as substrates (pH 5.0, 25°C); error bars show standard deviations from triplicate measurements

	ABTS			DMP		
Enzyme	k_cat_ (s^−1^)	K_m_(μM)	k_cat_/K_m_ (s^−1^ mM^−1^)	k_cat_ (s^−1^)	K_m_(μM)	k_cat_/K_m_ (s^−1^ mM^−1^)
Lac	5.2 ± 0.2	23.3 ± 3.4	222.8 ± 6.7	2.8 ± 0.5	46.1 ± 4.6	60.0 ± 3.8
LacPG	5.0 ± 0.4	56.0 ± 5.6	89.8 ± 4.5	2.5 ± 0.9	74.6 ± 8.0	33.6 ± 1.4
