# Supplementary material for: Ionic Polymer-Coated Laccase with High Activity and Enhanced Stability: Application in the Decolourisation of Water Containing AO7
Source: Sci Rep. 2015 Feb 5;5:8253. doi: 10.1038/srep08253 (PMC4317704; doi:10.1038/srep08253)
Supplement: Supplementary Information [file srep08253-s1.pdf]

Supplementary information

Ionic Polymer-Coated Laccase with High Activity and Enhanced Stability: Application in the Decolourisation of Water Containing AO7

Xiaolin Zhang, Ming Hua, Lu Lv & Bingcai Pan*

State Key Laboratory of Pollution Control and Resource Reuse, School of the Environment, Nanjing University, Nanjing 210023, P.R. China

*To whom correspondence should be addressed

E-mail: bcpan@nju.edu.cn (B.C.P.)

Tel: +86-25-8968-0563

The *Supplementary Information Available* contains 10 pages, including Figures S1-S9.

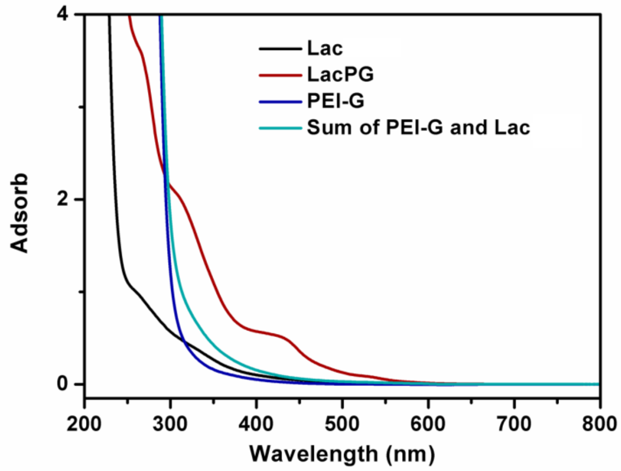

Figure S1. UV-vis spectra of Lac, LacPG, PEI-G dissolved in phosphate buffer solutions (pH 7.0, 0.05 M); the concentrations of corresponding components were: 1 mg/mL laccase protein, 0.05% PEI (w/v) and 0.05% GA (v/v).

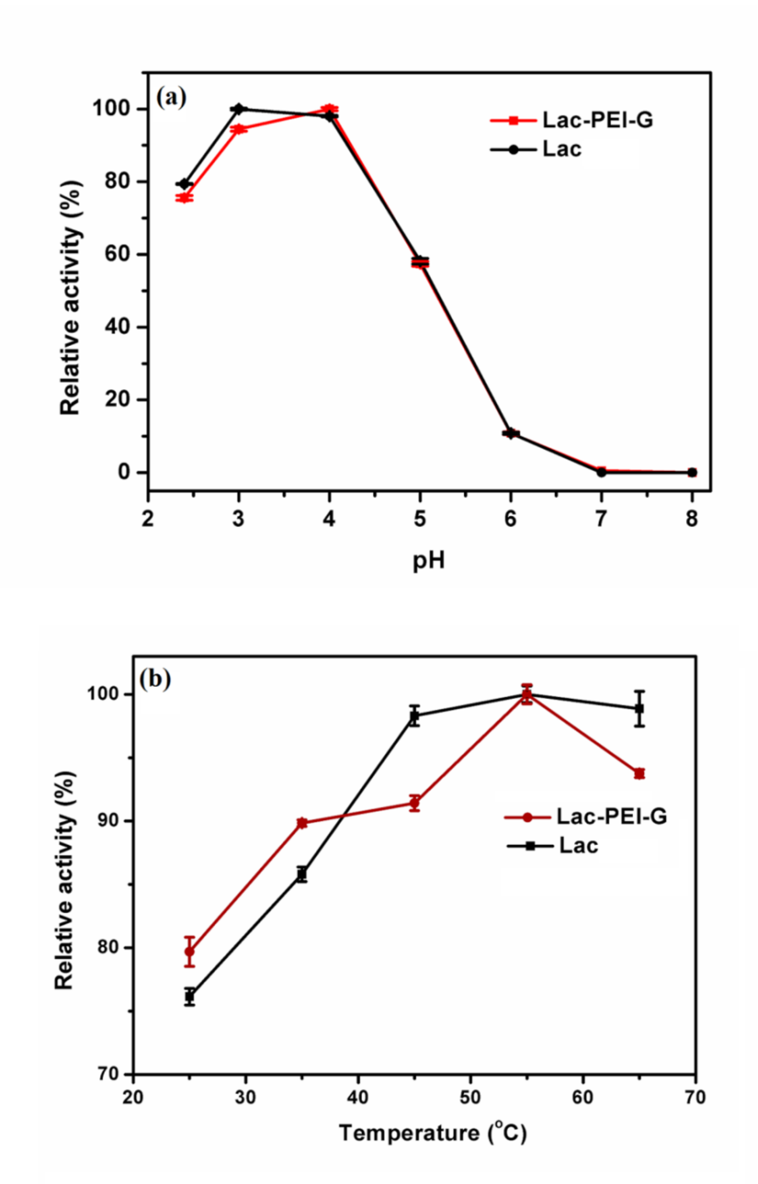

Figure S2. The effects of pH (a, at 25 ^{o}C) and temperature (b, at pH 5.0) on the activity of Lac and LacPG; error bars represents standard deviations from triplicate measurements**.**

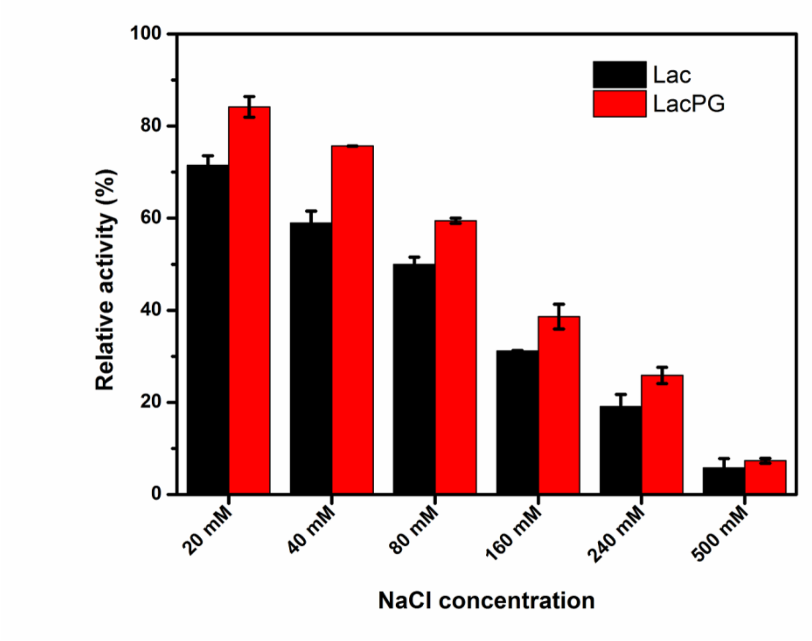

Figure S3. The effects of the concentration of chloride ions on the activity of Lac and LacPG; error bars represents standard deviations from triplicate measurements**.**

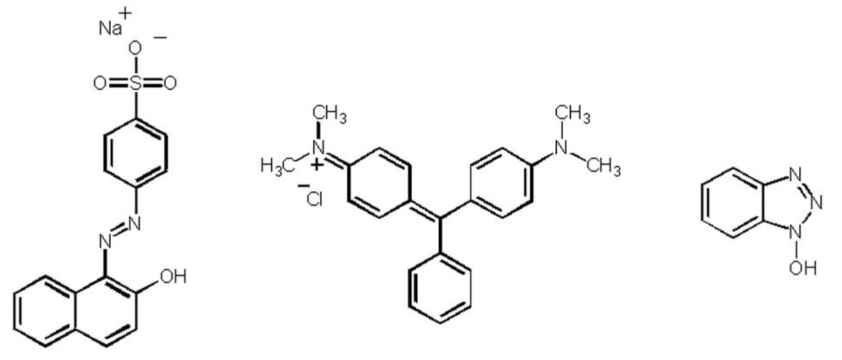

AO7 **MG** **HBT**

Figure S4. The schematic structures of AO7, MG and HBT in this study

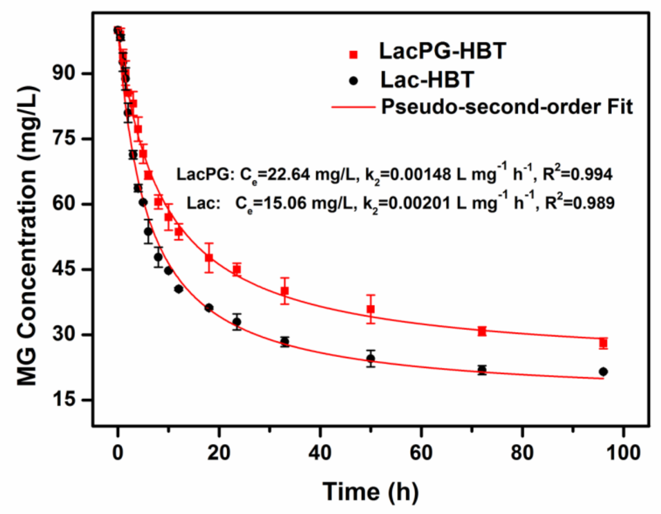

Figure S5. Decolourisation of MG using Lac and LacPG in presence of HBT (pH 5.0, 25 ℃); error bars represent standard deviations from triplicate measurements.

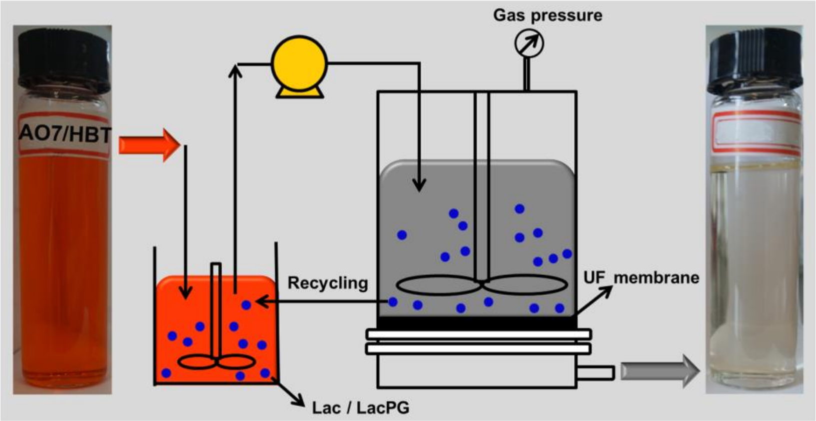

Figure S6. Schematic illustration of the enzymatic membrane reactor for cyclic AO7 decolourisation.

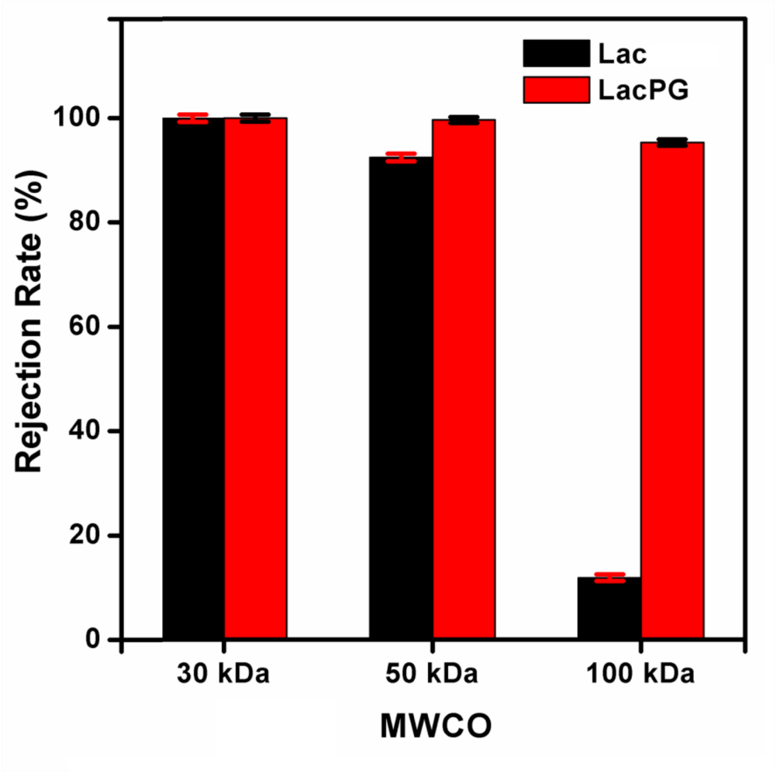

Figure S7. The rejection of Lac and LacPG separated by ultrafiltration membrane with different MWCO (25 ℃, pressure=0.1 MPa)

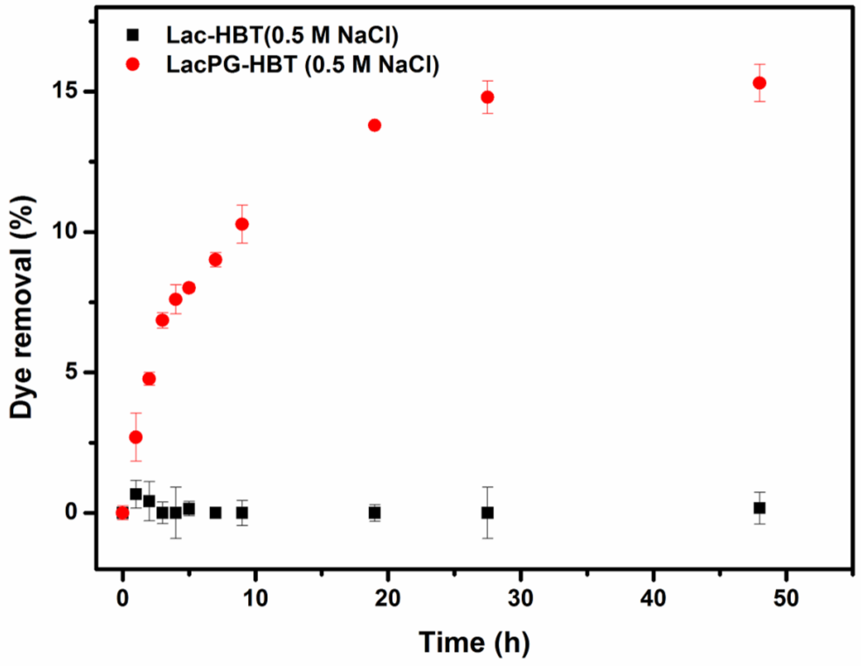

Figure S8. Decolourisation of AO7 containing 0.5 M NaCl using Lac and LacPG in presence of HBT (pH 5.0, 25 ℃); error bars represent standard deviations from triplicate measurements.

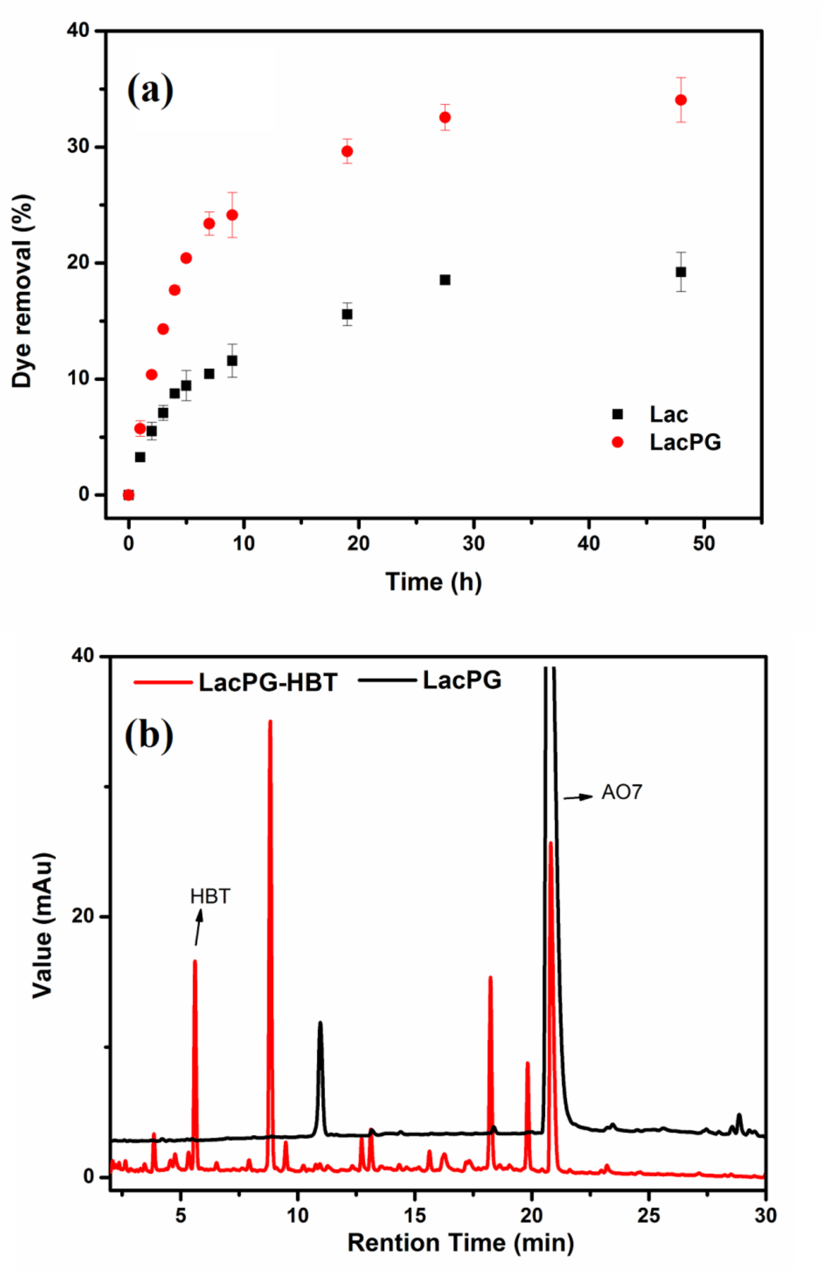

Figure S9. Decolourisation of AO7 using Lac and LacPG in presence and in absence of HBT (pH 5.0, 25 ℃) (a) and metabolite of AO7 degraded by LacPG in presence and in absence of HBT monitored by HPLC at 254 nm (b); error bars represent standard deviations from triplicate measurements.
